# Development and Comparison of Visual LAMP and LAMP-TaqMan Assays for *Colletotrichum siamense*

**DOI:** 10.3390/microorganisms12071325

**Published:** 2024-06-28

**Authors:** Shuning Cui, Haoze Ma, Xinyue Wang, Han Yang, Yuanzheng Wu, Yanli Wei, Jishun Li, Jindong Hu

**Affiliations:** Ecology Institute, Qilu University of Technology (Shandong Academy of Sciences), Jinan 250013, China; 13335151750@163.com (S.C.); mhz012174@163.com (H.M.); wangxinyue9908@163.com (X.W.); hanyang199905@163.com (H.Y.); wuyzh@sdas.org (Y.W.); weiyl@sdas.org (Y.W.); yewu2@sdas.org (J.L.)

**Keywords:** *Colletotrichum siamense*, loop-mediated isothermal amplification, pathogen detection

## Abstract

Strawberry anthracnose caused by *Colletotrichum* spp. has resulted in significant losses in strawberry production worldwide. Strawberry anthracnose occurs mainly at the seedling and early planting stages, and *Colletotrichum siamense* is the main pathogen in North China, where mycelia, anamorphic nuclei, and conidia produced in the soil are the main sources of infection. The detection of pathogens in soil is crucial for predicting the prevalence of anthracnose. In this study, a visualized loop-mediated isothermal amplification (LAMP) assay and a loop-mediated isothermal amplification method combined with a TaqMan probe (LAMP-TaqMan) assay were developed for the β-tubulin sequence of *C. siamense*. Both methods can detect *Colletotrichum siamense* genomic DNA at very low concentrations (10^4^ copies/g) in soil, while both the visualized LAMP and LAMP-TaqMan assays exhibited a detection limit of 50 copies/μL, surpassing the sensitivity of conventional PCR and qPCR techniques, and both methods showed high specificity for *C. siamense*. The two methods were compared: LAMP-TaqMan exhibited enhanced specificity due to the incorporation of fluorescent molecular beacons, while visualized LAMP solely necessitated uncomplicated incubation at a constant temperature, with the results determined by the color change; therefore, the requirements for the instrument are relatively straightforward and user-friendly. In conclusion, both assays will help monitor populations of *C. siamense* in China and control strawberry anthracnose in the field.

## 1. Introduction

The strawberry (*Fragaria ananassa*) is globally renowned for its delectable fragrance, vibrant red hue, and succulent texture, as well as its rich reservoir of bioactive compounds and dietary fibers. Moreover, this high-value fruit crop plays a pivotal role in agriculture and local economies worldwide [1]. According to the United Nations Food and Agriculture Organization (FAO), China’s strawberry planting area in 2022 reached 127,295 ha, with a total yield of 3.36 million tons, accounting for approximately 32.02% of the global strawberry cultivation area and 35.15% of the output. However, the frequent occurrence of strawberry fungal diseases in China is primarily attributed to the prevailing high temperature and humidity conditions within strawberry facilities, which significantly impede both the yield and quality of strawberries. The fungal disease anthracnose, primarily caused by *Colletotrichum* spp., manifests as black or dark brown sunken lesions on the surfaces of leaves or fruits. In infected fruits, these lesions can generate a substantial quantity of conidia [2]. Currently, a total of 257 *Colletotrichum* species, grouped into 15 complexes and 13 additional singletons, have been accepted in this genus [3], which has been reported to infect more than 3000 species of herbaceous and woody crops [4] and ranks among the top ten most important fungal pathogens worldwide [5], leading to substantial crop losses on a global scale. Strawberry anthracnose, one of the diseases affecting strawberries, is characterized by lesions on petioles and leaf whorls, as well as irregular blotches on leaves and black spots on fruits. Eventually, the entire plant wilts and dies [6]. The infestation of strawberry anthracnose occurs from the seedling stage, with severe impacts on leaves and stolons, leading to frequent outbreaks during the middle and late stages. In severe cases, it can result in a yield reduction exceeding 50%. So far, the anthracnose epidemic has affected strawberry nurseries and production fields worldwide and is one of the most destructive diseases affecting strawberry production worldwide. *C. siamense* is the most predominantly reported (45.9% of the total) species in the strawberry fields of China across a wide range of latitudes and is distributed in nearly all provinces except for Liaoning [7]. *C. siamense* is classified in the *Colletotrichum gloeosporioides* complex [8]. The pathogenicity of *C. siamense* has been confirmed as the primary cause of anthracnose and stem rot in strawberry cultivation in northern China [9,10,11].

Anthracnose is characterized by a long incubation period, a concentrated onset, and strong outbreaks [12]. Therefore, early detection of anthracnose is of paramount importance. In recent years, the loop-mediated isothermal amplification (LAMP) assay has gained widespread recognition for its application in the molecular diagnosis of plant diseases [13]. In comparison to conventional PCR, LAMP enables rapid and specific detection of DNA through the use of four primers targeting six sequences at a constant temperature [14]. Furthermore, the incorporation of the loop primers LF and LB not only accelerates the LAMP reaction but also enhances its sensitivity [15]. Initially, LAMP amplification products were detected using agarose gel electrophoresis. However, the presence of high concentrations of nucleic acids in the reaction products poses a risk of aerosol contamination upon opening the centrifuge tubes. Recently, innovative approaches have been developed to visualize results without exposing them to potential contamination risks. These methods involve incorporating specific indicators that induce color changes before and after the reaction, such as the turbidity methods [16], metal ion indicator methods [17], pH indicator methods [18], fluorescence methods [19], etc. Due to primer self-association, system contamination, and other reasons, the LAMP technique exhibits a relatively low probability of nonspecific amplification and false positives [20]. Nevertheless, there are still certain limitations in the practical application of LAMP.

To address these issues, fluorescent molecular beacon techniques have been used to directly validate the amplification process [21]. One of the common fluorescent molecular beacon assays involves the use of TaqMan probes, whose 5′ terminal fluorescent groups are quenched by the 3′ terminal groups when they are not hybridized to the target sequence. Upon binding to its target sequence, the probe undergoes cleavage of the 5′-terminal fluorescent group by a DNA polymerase with 5′-3′ endonuclease activity, resulting in separation from the quenched group and generation of a fluorescent signal [22]. TaqMan probes have also been successfully used in LAMP; similar to qPCR, Bst5.1 DNA polymerase with 5′-3′ endonuclease activity is utilized in the LAMP system [23]_,_ or TaqDNA polymerase is added to the reaction system [24], which generates a fluorescent signal during the amplification process and detects the amplification results by monitoring the fluorescent signal on a thermal cycler. Since this method relies on the fluorescent signal generated by the cleavage of the fluorescent group of the probe to detect successful amplification, the LAMP-TaqMan method effectively mitigates nonspecific amplification and false positives, thereby enhancing the accuracy of the results.

In this study, we developed a visually enhanced loop-mediated isothermal amplification assay and a TaqMan probe-incorporated loop-mediated isothermal amplification method (LAMP-TaqMan) for the detection of *Colletotrichum siamense*. The visualized loop-mediated isothermal amplification assay utilized the pH indicator method to determine the amplicon, while LAMP-TaqMan incorporated Taq DNA polymerase and a TaqMan probe. This study allows for new experiments in the field and fills the research gap for the rapid detection of the strawberry anthracnose pathogen *C*. *siamense*. The present study aimed to evaluate and compare the two assays with the objective of providing technical support for real-time detection in field settings.

## 2. Materials and Methods

### 2.1. Strains

The pathogenic fungi used in the experiment were obtained from the Environmental Microbiology Laboratory of the Institute of Ecology, Shandong Academy of Sciences, China (Table 1). All strains were grown on PDA agar plates for 7 d. Genomic DNA was extracted and purified using a Fungal DNA Kit (Magen), and the DNA was stored at −20 °C for subsequent use. The genomic DNA was quantified using the relative quantification tool in Image Lab (Hercules, CA, USA).

### 2.2. Visualization of LAMP and LAMP-TaqMan Primer Design

The conserved regions within the β-tubulin sequence of *Colletotrichum siamense* were used as the target DNA fragment for LAMP. The β-tubulin homologous sequences of the same species and genus were downloaded from the NCBI website for comparative analysis, while the ESPript3 website (https://espript.ibcp.fr/ESPript/ESPript/index.php accessed on 30 May 2024) was utilized to compare multiple sequences and select specific primers targeting nonhomologous regions. LAMP primers were designed using the NEB LAMP Primer Design Tool (https://lamp.neb.com/#! accessed on 30 May 2024), and the probe for LAMP-TaqMan and TaqF was designed using the Premier program (Primer Premier 5). The specificity of all primers was initially validated by primer-BLAST (https://blast.ncbi.nlm.nih.gov/Blast.cgi accessed on 30 May 2024), and the primers were synthesized by Sango BioTech (Shanghai, China).

### 2.3. Visualizing LAMP and Conditional Optimization

The visual LAMP reaction was conducted in a total volume of 20 µL, which included 0.2 μM F3/B3, 1.6 μM FIP/BIP, 0.4 μM LF/LB, 50 mM KCl, 10 mM (NH_4_)_2_SO_4_, 0.1% *v*/*v* Tween-20, 2.8 mM dNTPs, 8 mM MgSO_4_, 0.4 M betaine, 200 mM cresol red, a buffer system adjusted to a pH of 8.6–8.8 (adjusted with 1 M KOH), 40 U of Bst DNA plus polymerase, and 1 μL of DNA template.

To determine the optimal amplification temperature and duration, a gradient PCR instrument was used to perform a gradient reaction at 60–65 °C for 30–80 min, followed by inactivation at 85 °C for 5 min. The positive samples were bright yellow, while the negative samples were rose red at the end of the reaction. The obtained results were subsequently validated through electrophoresis on a 2% agarose gel at 120 V for 30 min.

### 2.4. LAMP-TaqMan System Assay and Condition Optimization

The LAMP-TaqMan assay was carried out in a reaction mixture (with a final volume of 20 μL) containing 0.2 μM F3/B3, 1.6 μM FIP/BIP, 0.4 μM LF/LB/TaqF, 2 μL of 10× Hieff^®^ Bst Plus DNA Polymerase Buffer, 2.8 mM dNTPs, 8 mM MgSO_4_, 0.8 M betaine, 40 U of Bst DNA plus polymerase, 2 U of Taq DNA polymerase, 200 μM CS probe, and 1 μL of DNA template. The LAMP-TaqMan reaction was performed using a real-time PCR system (Bio-Rad cfx90, Hercules, CA, USA), and to determine the optimal amplification temperature, a gradient reaction ranging from 55 °C to 68 °C was performed for 60 min, followed by inactivation at 85 °C for 5 min. During incubation at a constant temperature, the FAM fluorescence signal was generated by quantifying the fluorescent moiety of the probe with a fluorescence detector. Subsequently, a real-time amplification curve was constructed and processed to accurately estimate and distinguish positive results. After the procedure, the Bio-Rad CFK manager was used to generate amplification curves for subsequent analysis.

### 2.5. Specificity Detection and Sensitivity Analysis of LAMP and LAMP-TaqMan

The conserved region of the β-tubulin gene in *C. siamense* was amplified using the primers CS-F3/CS-B3, and a pMD-18T vector kit was used to construct the standard plasmid pMD-18T-CS. The standard plasmid was sequenced by Sango BioTech (Shanghai, China), while quantification of the plasmid was performed using the ds DNA Quantification Kit (Shanghai BioTech, Shanghai, China).

The conidia of *C. siamense* were diluted sequentially 10-fold to a concentration of 1 × 10^2^ CFU/mL, starting from an initial concentration of 1 × 10^8^ CFU/mL. Subsequently, 25 μL of the conidial suspension was mixed with sterilized soil (0.25 g) to prepare inoculated conidial concentrations ranging from 1 × 10^7^ CFU/g to 10 CFU/g in the soil samples. Soil DNA extraction was performed using the DNeasy^®^ PowerSoil^®^ DNA Kit (QIAGEN, Hilden, Germany).

To determine the specificity of visualized LAMP, the LAMP-TaqMan assay was used for *C. siamense* detection using various DNA samples of different pathogens (Table 1) as templates. To compare the sensitivity of *C. siamense* detection using visual LAMP and LAMP-TaqMan, different concentrations of the *C. siamense* standard plasmid pMD-18T-CS and soil DNA inoculated with *C. siamense* conidia were used as templates for amplification. For the plasmid assay, a 10-fold serial dilution was performed on the standard plasmid concentration of *C. siamense* ranging from 5 × 10^6^ copies/μL to 5 copies/μL, while for the soil assay, soil DNA with spore concentrations ranging from 10^7^ CFU/g to 10^2^ CFU/g was used as a template to determine the detection limits of the visual LAMP and LAMP-TaqMan assays. Ultrapure water was used as a template for the negative control, and all assay tests were repeated three times.

### 2.6. Application of Testing to Real Samples

To evaluate the practical applicability of the LAMP and LAMP-TaqMan assays developed in this study, a total of 20 strawberry samples were collected from Jinan, Shandong Province, and 38 soil samples obtained from strawberry fields were employed for testing. The DNA extraction method used for strawberry samples was CTAB-based, whereas the DNeasy^®^ PowerSoil^®^ DNA Kit (QIAGEN) was utilized for soil sample extraction.

## 3. Results

### 3.1. Visualization of LAMP and LAMP-TaqMan Primer Design

**Figure 1:**
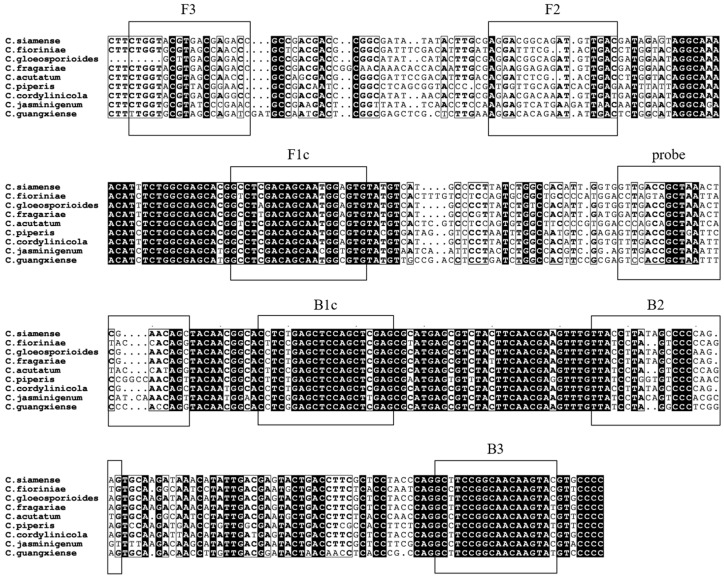
Schematic diagram of the β-tubulin gene alignment of 9 *Colletotrichum* species.

The β-tubulin homologous sequences of the various species of *Colletotrichum* were downloaded for comparative analysis in order to select specific primers targeting nonhomologous regions. LAMP primers were designed using the NEB LAMP Primer Design Tool (https://lamp.neb.com/#! accessed on 30 May 2024) (Figure 1, Table 2). Both LAMP and LAMP-TaqMan were amplified using these LAMP primers. In LAMP-TaqMan, we introduced the primer TaqF at the 5′ end of the probe to enhance the efficiency of the LAMP-TaqMan fluorescence reaction.

LAMP amplification in the *C. siamense* assay could produce a multiplicative gradient sequence from the F2 primer to the R2 primer (approximately 206 bp). Primer-BLAST analysis revealed that the designed primers exhibited exclusive amplification specificity toward *C. siamense* and no predicted nonspecific amplification products.

### 3.2. Optimization and Validation of the Visual LAMP Assay and LAMP-TaqMan Assay

**Figure 2:**
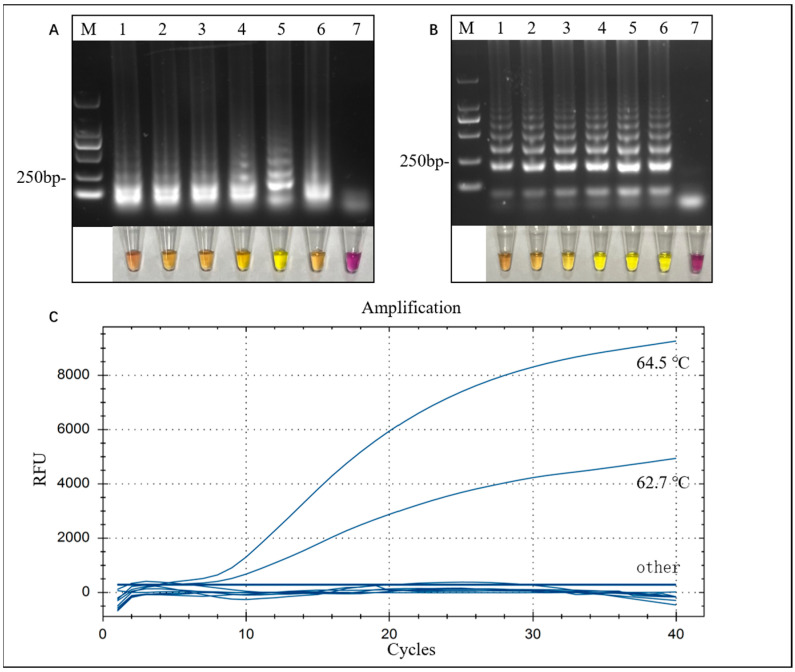
Optimization of the visual LAMP and LAMP-TaqMan assays. (**A**) Optimization of the visual LAMP reaction temperature. M is the DL2000 bp marker; numbers 1–6 represent samples amplified at 60 °C, 61 °C, 62 °C, 63 °C, 64 °C, and 65 °C, respectively; number 7 is a negative control. Below is the system color corresponding to the lanes. (**B**) Optimization of the visual LAMP reaction time. M is the DL2000 bp marker; numbers 1–6 represent samples with 30 min, 40 min, 50 min, 60 min, 70 min, and 80 min reaction times, respectively; number 7 is the negative control. Below is the system color corresponding to the lane. (**C**) Optimization of the LAMP-TaqMan reaction temperature.

Visual LAMP results and optimization were determined based on visual color changes and further validated through agarose gel electrophoresis. The temperature and time of the visual LAMP reaction were optimized, and the optimized reaction temperature is shown in Figure 2A. When the LAMP reaction mixture was incubated for 60 min at the designated temperature range, the system became yellow when amplified at 64 °C, and the amplification system became orange-yellow at other temperatures. No color change was observed in the control group. The results showed that amplification at 64 °C produced the highest copy number and exhibited the most obvious visualization results; the visual LAMP results were further validated through agarose electrophoresis, and the optimal amplification conditions were determined based on the brightness of the amplified bands. The LAMP assay yielded distinct and intense bands when conducted at a temperature of 64 °C. The optimization of the reaction time is shown in Figure 2B. The LAMP reaction mixture was incubated at this optimized temperature for 30–80 min, and the system exhibited a yellow hue, while the color of the control group remained unchanged. At the beginning, as the reaction proceeded, the visualized LAMP system displayed an intensified yellow color; after 60 min of reaction, the color of the system did not change with time. The above results demonstrate that a 60 min amplification at 64 °C represents the optimal condition for visual LAMP.

The LAMP-TaqMan results were determined by the continuous generation of fluorescent signals, as shown in Figure 2C. The continuous fluorescence signals appeared earliest and exhibited the highest intensity when the amplification was performed at 64.5 °C, indicating that 64.5 °C was the optimal amplification condition for LAMP-TaqMan.

### 3.3. Specificity and Sensitivity of the Visual LAMP and LAMP-TaqMan Assays

**Figure 3:**
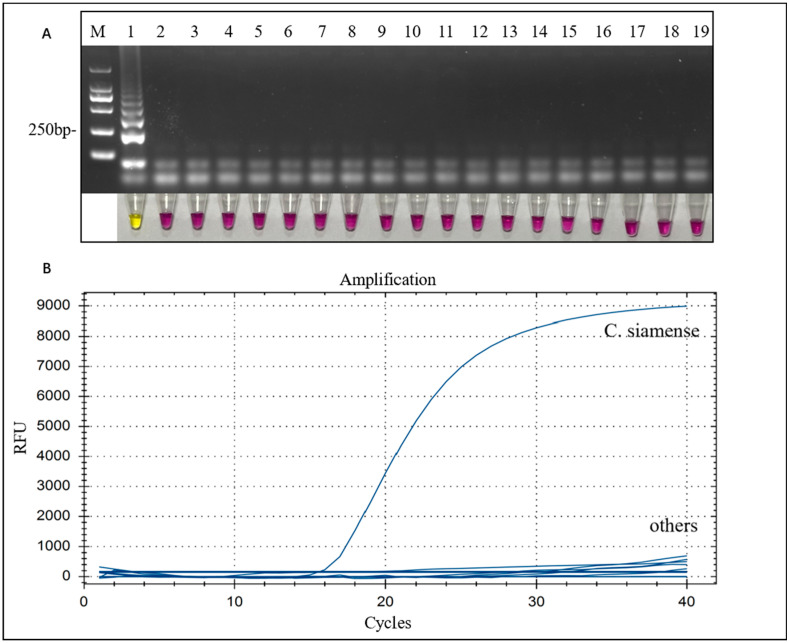
Specificity of visual LAMP and LAMP-TaqMan to *C. siamense*. Assessment is based on (**A**) gel electrophoresis analysis of the LAMP products and cresol red-visualized color change. Lane M is the DL2000 marker, the templates of lanes 1–18 represent samples with different amounts of pathogenic bacterial DNA, as shown in Table 1, and lane 19 is a negative control. The corresponding cresol red-visualized system color changes are shown below. (**B**) Fluorescence curves for LAMP-TaqMan specificity detection.

Specific assays were performed using the genomic genes of 18 strains (Table 1) of pathogenic bacteria conserved from the laboratory as templates, and ddH_2_O was used as a negative control template. The results showed that only the *C. siamense* sample exhibited successful amplification in both the visual LAMP and LAMP-TaqMan assays. In the visual LAMP assay, this was indicated by a color change from red to yellow, which was further confirmed through agarose gel electrophoresis (Figure 3A). Similarly, in the LAMP-TaqMan assay, fluorescence changes were detected along with the appearance of an amplification curve (Figure 3B).

**Figure 4:**
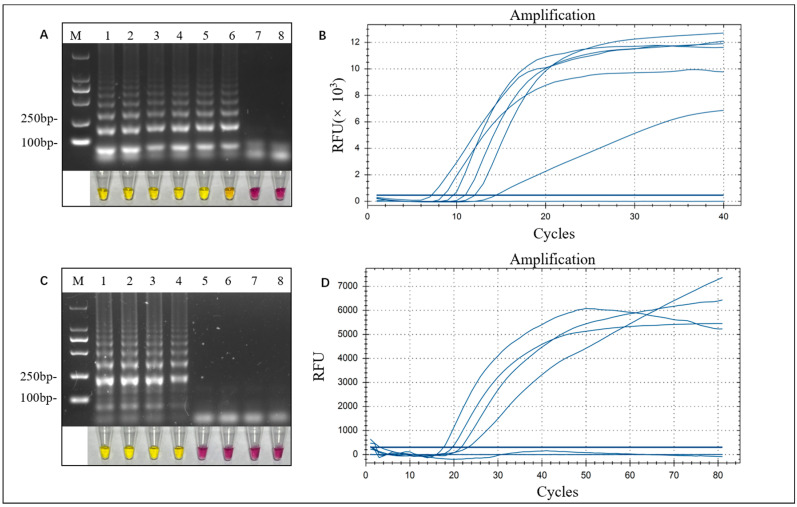
Sensitivity of visual LAMP and LAMP-TaqMan for the detection of *C. siamense*. (**A**) Gel electrophoresis and cresol red-visualized color change. Lane M is the DL2000 bp marker, and the concentrations of the standard plasmids in lanes 1–7 are 5 × 10^6^ copies/μL, 5 × 10^5^ copies/μL, 5 × 10^4^ copies/μL, 5 × 10^3^ copies/μL, 5 × 10^2^ copies/μL, 50 copies/μL, and 5 copies/μL, respectively; lane 8 is the negative control. (**B**) Fluorescence curves of LAMP-TaqMan are plotted based on the peak time fluorescence curves, arranged in ascending order of plasmid concentration: 5 × 10^6^ copies/μL, 5 × 10^5^ copies/μL, 5 × 10^4^ copies/μL, 5 × 10^3^ copies/μL, 5 × 10^2^ copies/μL, and 5 × 10^1^ copies/μL. (**C**) Gel electrophoresis and cresol red-visualized color change using the soil genome as a template. Lane M is the DL2000 bp marker, and the final spore concentrations of the soil in lanes 1–6 are 10^7^ CFU/g, 10^6^ CFU/g, 10^5^ CFU/g, 10^4^ CFU/g, 10^3^ CFU/g, and 10^2^ CFU/g, respectively; lane 7 is the uninoculated spore soil DNA, and lane 8 is the negative control. (**D**) Fluorescence curves of LAMP-TaqMan. The concentration of spores in the template ranged from 10^7^ CFU/g on the left to 10^4^ CFU/g on the right, following a decreasing order of magnitude.

LAMP and LAMP-TaqMan reactions were performed under optimized conditions using different concentrations of the standard plasmid pMD-18T-CS and soil DNA inoculated with *C. siamense* spores as templates. Visual LAMP was successfully amplified when the standard plasmid template was diluted to 50 copies/μL (Figure 4A), whereas the standard plasmid template required 500 copies/μL for successful PCR amplification. Thus, visual LAMP was 10 times more sensitive than PCR. The results indicated that the detection sensitivity of the LAMP-TaqMan assay was also limited to 50 copies/μL of standard plasmid (Figure 4B).

In the inoculated spore soil assay, visual LAMP and LAMP-TaqMan enabled the detection of soil DNA at inoculum concentrations greater than 10^4^ CFU/g of *C. siamense* spores (Figure 4C,D). In summary, both the visual LAMP reaction and LAMP-TaqMan could detect lower levels of *C. siamense.*

### 3.4. Application of Testing to Real Samples

The results for 20 strawberry samples and 38 soil samples are shown in Table 3. In the soil samples, a total of three positive samples were detected by visualized LAMP, and four positive samples were detected by LAMP-TaqMan. In the strawberry samples, both visualized LAMP and LAMP-TaqMan detected three positive samples. The agreement between the two methods was 98.28%. Both methods can be used for detecting *C. siamense.*

## 4. Discussion

Accurate detection is a crucial component of early anthracnose management. It facilitates the adoption of appropriate control strategies and technical solutions prior to symptom manifestation. In this study, we developed a visual LAMP assay based on colorimetric changes and a LAMP-TaqMan assay utilizing fluorescence detection for rapid and precise identification of *C. siamense* in planted soil.

In this study, the variable region of the *C. siamense* β-tubulin gene was selected as the target for primer design. For the visual LAMP assay, the optimal reaction conditions were 64 °C for 60 min, and the reaction system was weakly buffered. During nucleic acid amplification, a significant amount of pyrophosphate and hydrogen ions were released as byproducts, resulting in a pH decrease of 2–3 units at the end of the reaction. The activity of DNA polymerase and DNA amplification remained unaffected by the buffer-free system and pH variations [25]. Cresol red was employed as the pH indicator in this study and exhibited a distinct color change before and after the reaction. The optimal fluorescence curve in the LAMP-TaqMan assay was obtained by performing the reaction at 64.5 °C. In addition to the LAMP primer and the loop primer, LAMP-TaqMan also introduced the primer TaqF, which facilitated the precise cleavage of the probe at its 5′ end by TaqDNA polymerase, leading to the release of the fluorescence signal, and the fluorescence was observed approximately ten min earlier in the presence of TaqF. The TaqMan probe was strategically positioned between the F3 and B3 primers within the variable region, thereby ensuring its specificity and enhancing its suitability for experimental purposes.

In previous studies, a real-time fluorescent quantitative PCR assay was developed for the detection of *C. siamense* [26], with a sensitivity of 1 ng/μL of *C. siamense* DNA (approximately 1.5 × 10^4^ copies/μL). In the detection of other species in the genus *Colletotrichum*, assays have been developed for *C. gloeosporioides* [27], *C. truncatum* [28], *C. acutatum* [29], and other species. The LAMP assay demonstrated a remarkable detection limit as low as 10^−4^ ng/μL [30]. From the results of this study, both visual LAMP and LAMP-TaqMan achieved detection limits of 50 copies/μL, which is one-thousandth of that of real-time fluorescence quantitative PCR and similar to other LAMP assays on *Colletotrichum* spp. The assay exhibited exceptional detection sensitivity, fully satisfying the testing requirements for *C. siamense*. In the specificity test of the assay, the two assays could distinguish *C. siamense*, *C. gloeosporioides*, and *C. fragariae* from three closely related *Colletotrichum* spp., and other pathogenic fungi were isolated from the strawberry samples; no amplification was detected.

The visual LAMP and LAMP-TaqMan methods were verified by testing strawberry samples and soil samples in the field. The results obtained from both detection methods exhibited a high degree of concordance. Furthermore, an additional positive sample was detected using the LAMP-TaqMan method during the analysis of the soil samples. The sensitivity of the LAMP-TaqMan assay was superior to that of the visual LAMP assay in practical applications, and both methods demonstrated efficacy for detecting *C. siamense.*

In a subsequent study, the correlation between the results of visual LAMP and LAMP-TaqMan assays and the incidence of strawberry disease was analyzed by potting strawberries in soil inoculated with different concentrations of *C. siamense* conidia at different time points. The experimental results showed that strawberries were susceptible to disease when exposed to spore concentrations exceeding 10^4^ CFU/g in the soil. Both visual LAMP and LAMP-TaqMan assays detected positive results on the leaves of plants exhibiting soil spore concentrations exceeding 10^4^ CFU/g on Day 14 after podding, while the plants did not show symptoms of disease at the time.

## 5. Conclusions

The visual LAMP and LAMP-TaqMan assays enable rapid and precise detection of *Colletotrichum siamense* in strawberries and soil. The LAMP-TaqMan method employs the principles of LAMP and utilizes the TaqMan probe for fluorescence quantification, demonstrating comparable sensitivity and specificity to the visual LAMP method while exhibiting a higher detection limit than that of qPCR. Therefore, implementing preventive and control measures before *Colletotrichum siamense* spreads in agricultural settings would be helpful.

## Figures and Tables

**Table 1:** Fungal strains used in the experiment.

Number	Fungal Species	Isolate
1	*Colletotrichum siamense*	CM9
2	*Colletotrichum gloeosporioides*	GJ4
3	*Colletotrichum fragariae*	CM10
4	*Mucor irregularis*	G121
5	*Mucor circinelloides*	G122
6	*Fusarium solani*	T1X2
7	*Fusarium oxysporum*	T1X3
8	*Acremonium egyptiacum*	T6T3
9	*Botrytis cinerea*	G61
10	*Didymella bellidis*	T1X32
11	*Dactylonectria macrodidyma*	T1X4
12	*Paraconiothyrium brasiliense*	T6T2
13	*Trichoderma atroviride*	HB20111
14	*Botryosphaeria dothidea*	Bd5
15	*Alternaria alternata*	d1
16	*Rizoctonia solani*	Rs21
17	*Fusarium pseudograminearum*	Fp30
18	*Fusarium equiseti*	Fe9

**Table 2:** Sequences of *Colletotrichum siamense*-specific LAMP primers and the TaqMan probe.

Primer Name	Sequence 5′-3′	Length (bp)
CS-F3	CTGGTACGTGACGAGACC	18
CS-B3	GTACTTGTTGCCGGAAGC	18
CS-FIP	CACTCCATTGCTGTCGAGGCGAGGACGGCAGATGTTGA	38
CS-BIP	CCTCTGAGCTCCAGCTCGAGCTCTGGGGGGCTATAAGGTAA	41
CS-LF	GCTCGCCAGAAATGTTTTGCCTA	23
CS-LB	CGCATGAGCGTCTACTTCAACG	22
CS-probe	GTTGACCGCTAAACTCGAACAGC	23
CS-TaqF	AAACATTTCTGGCGAGCACG	20

**Table 3:** Visual LAMP and LAMP-TaqMan detection of *C. siamense* in plant tissues and soil.

Sample Name	Sample Size	LAMP		LAMP-TaqMan	
		Positive Sample	Negative Sample	Positive Sample	Negative Sample
Soil samples	38	3	35	4	34
Strawberry Sample	20	3	17	3	17

## Data Availability

All data are contained within the article.
